# Impact of Age, Caloric Restriction, and Influenza Infection on Mouse Gut Microbiome: An Exploratory Study of the Role of Age-Related Microbiome Changes on Influenza Responses

**DOI:** 10.3389/fimmu.2017.01164

**Published:** 2017-09-20

**Authors:** Jenna M. Bartley, Xin Zhou, George A. Kuchel, George M. Weinstock, Laura Haynes

**Affiliations:** ^1^UConn Center on Aging, Farmington, CT, United States; ^2^Department of Immunology, UConn Health, Farmington, CT, United States; ^3^Jackson Laboratory for Genomic Medicine, Farmington, CT, United States; ^4^Department of Genetics and Genome Sciences, UConn Health, Farmington, CT, United States

**Keywords:** aging, influenza, gut microbiome, caloric restriction, cytokines

## Abstract

Immunosenescence refers to age-related declines in the capacity to respond to infections such as influenza (flu). Caloric restriction represents a known strategy to slow many aging processes, including those involving the immune system. More recently, some changes in the microbiome have been described with aging, while the gut microbiome appears to influence responses to flu vaccination and infection. With these considerations in mind, we used a well-established mouse model of flu infection to explore the impact of flu infection, aging, and caloric restriction on the gut microbiome. Young, middle-aged, and aged caloric restricted (CR) and ad lib fed (AL) mice were examined after a sublethal flu infection. All mice lost 10–20% body weight and, as expected for these early time points, losses were similar at different ages and between diet groups. Cytokine and chemokine levels were also similar with the notable exception of IL-1α, which rose more than fivefold in aged AL mouse serum, while it remained unchanged in aged CR serum. Fecal microbiome phyla abundance profiles were similar in young, middle-aged, and aged AL mice at baseline and at 4 days post flu infection, while increases in Proteobacteria were evident at 7 days post flu infection in all three age groups. CR mice, compared to AL mice in each age group, had increased abundance of Proteobacteria and Verrucomicrobia at all time points. Interestingly, principal coordinate analysis determined that diet exerts a greater effect on the microbiome than age or flu infection. Percentage body weight loss correlated with the relative abundance of Proteobacteria regardless of age, suggesting flu pathogenicity is related to Proteobacteria abundance. Further, several microbial Operational Taxonomic Units from the Bacteroidetes phyla correlated with serum chemokine/cytokines regardless of both diet and age suggesting an interplay between flu-induced systemic inflammation and gut microbiota. These exploratory studies highlight the impact of caloric restriction on fecal microbiome in both young and aged animals, as well as the many complex relationships between flu responses and gut microbiota. Thus, these preliminary studies provide the necessary groundwork to examine how gut microbiota alterations may be leveraged to influence declining immune responses with aging.

## Introduction

Aging is a complex process that has dramatic impacts on most systems in the body (1). This is especially true of the immune system where significant age-related changes are observed in both innate and adaptive immune responses. One of the most prominent manifestations of aging is an increase in susceptibility to infections. In fact, influenza infection is one of the top killers of elderly people in the world, with the oldest being most at risk (2, 3). In aging mouse models, the clearance of influenza virus is slower and T cell responses are reduced and delayed when compared to young mice, which closely mirrors what happens during influenza infection of elderly humans (4–7). In addition, our recent study demonstrated that there are lingering inflammatory cytokines such as IL-6, IL-1α, and G-CSF in the bronchiolar lavage fluid (BAL) of aged mice during influenza infection. We have also shown that there is an increasing level of albumin in the BAL of aged mice, which is indicative of lung damage during the later stages of infection (8). These results indicate that the inability to efficiently clear virus in aged lungs corresponds to extended inflammation and lung damage.

One of the most consistent ways to delay aging in mice is *via* caloric restriction (CR). Indeed, CR was first shown to extend the lifespan of rodents in the 1930s (9) and has since been observed across multiple species, ranging from invertebrates to rodents to even some non-human primate studies (10–13); however, it is important to note that this is not observed in all studies (14–19) suggesting some mechanisms of longevity with CR may not be conserved among species and that details of implementation likely effect outcomes. Despite these discrepancies, and more importantly, along with extending lifespan, CR has been shown to delay age-related deficits in multiple physiological systems in mice (20–25), seemingly targeting the process of aging itself. From a translational perspective, human CR studies assessing longevity are nearly impossible, and short term studies evaluating other healthspan measures are difficult to control and criticized for practicality (please refer to Ref. (26) for a recent review of human CR trials). Regardless of these limitations, results from both population studies (27), as well as the Comprehensive Assessment of Long-term Effects of Reducing Intake of Energy (28–30) and Caloric Restriction with Optimal Nutrition (31, 32) studies show benefits in some areas, albeit not in all aspects of CR in rodent trials, suggesting CR does hold value within human aging research. Moreover, the benefits to multiple different systems evident in rodent studies makes it an attractive avenue to pursue. It is important to note that CR in these cases is not malnutrition (although feed is generally reduced by 40% caloric content, it is fortified with micronutrients to prevent deficiencies), and it is normally introduced after mice have reached maturation. There are many hypotheses for the mechanism of action of CR, including modulation of glucose–insulin homeostasis, growth hormone axis, autophagy changes, and alteration of inflammatory pathways; however, it is likely many of these changes may act in concert to delay aging. Nonetheless, CR has consistently shown improvement in multiple facets of aging, including delaying immunosenescence (33–37). Most notably, CR attenuates the shift from naive to memory phenotype observed in T cells with aging (38) and maintains the proliferative capacity of T cells (39, 40). Despite these positive effects on the aged immune system, the effects of CR on immune responses to influenza with aging is not clear. Original studies by Effros et al. (41) demonstrated that CR could enhance the immune response in aged mice ameliorating age-related declines in T cell proliferation and antibody production in response to intraperitoneal immunization to influenza. Conversely, work from Gardner and colleagues (42–45) demonstrated increased severity of infection in aged CR mice with increased viral titers and mortality in response to intranasal influenza infection attributed to impaired NK cell responses (43, 44) and/or reduced energy reserves and lethal weight loss (46). However, in these studies, high doses of influenza were utilized with mortality even observed in young mice; thus, it is unclear how CR modulates the immune response to more sublethal doses of influenza.

More recently, the importance of the microbiome in regulating immune responses has been elucidated. The gut microbiota have regulatory effects on not only intestinal immunity but also systemic immune responses (47) and systemic T cell subset populations can be skewed by different microbiota predominance (48–51). Further, pulmonary immunity is directly affected by the gut microbiota with regards to both allergic airway (52) and infectious disease (53, 54) responses. Importantly, the gut microbiota plays a major role in mediating flu infection-related immune responses and is particularly crucial for respiratory tract DCs migration, T cell priming, cytokine secretion, and overall viral clearance (53). Dysbiosis of the gut microbiota induced by antibiotic administration during flu infection influences helper T cell responses and can negatively impact flu outcomes and recovery (55). In addition, influenza infection itself induces gut microbiota dysbiosis through type I interferons (IFN-I) favoring Proteobacteria overgrowth (56). Thus, the relationship between gut microbiota and influenza infection seems complex and integrated.

Furthering this line of thought, the gut microbiome also changes with age [recently reviewed in Ref. (57, 58)]. More specifically, there seems to be a general decrease in microbiota diversity with aging (59, 60), as well as an increase in Proteobacteria abundance and lower levels of Firmicutes (59–63). Also, a shift toward a more Bacteroidetes dominated microbiome was associated with frailty (61). It is unknown how these changes to gut microbiota with aging may influence immune responses. CR also impacts the microbiome with greater levels of Lactobacillus and other potential probiotics associated with longevity (64). But importantly, the influence of gut bacteria microbiota changes on age-related pathologies has yet to be determined. It is possible that gut microbiota changes with CR may be a potential mechanism of longevity and/or related to some of the “antiaging” effects evident in many murine studies.

It is known that different components of nutrition can affect the aged immune system [reviewed elsewhere (65)] and that prebiotic/probiotic supplementation may decrease the severity of infection in the elderly (66–69). Moreover, small-scale studies have indicated that specific probiotics and prebiotics may improve flu vaccine response in hospitalized elderly (70–73) suggesting that age-related alterations in gut microbiota may precipitate reduced flu responses in the elderly, and that this dysbiosis can be treated to improve immune responses. Indeed, depletion of specific gut microbiota through antibiotic treatment can negatively affect both DC (53) and T cell (53, 55) influenza immune responses, while the gut microbiota itself is affected by influenza infection through type I interferons (IFN-I) (56), thus the relationship between gut microbiota and influenza infection is bidirectional and warrants further investigation. Here, in this exploratory study, we sought to examine how CR, a known modulator of aging and gut microbiota, can influence influenza-induced gut microbiota changes and immune responses during acute influenza infection in young, middle, and aged C57BL/6 mice. We hypothesized that CR would protect aged mice from age-related gut microbiota changes and thus mitigate influenza-induced changes to gut microbiota and improve immune responses.

## Materials and Methods

### Mice

Young (5–6 months old), middle (9–10 months old), and aged (19–21 months old) caloric restricted (CR) and *ad libitum* (AL) C57BL/6 male mice from the National Institute on Aging caloric restricted rodent colony were obtained at least 6 weeks prior to experimentation to allow appropriate acclimation to our facility. CR mice were fed the NIH31 fortified diet with CR was initiated at 14 weeks of age at 10% restriction, increased to 25% restriction at 15 weeks, and to 40% restriction at 16 weeks where it was maintained throughout the life of the animal. AL mice were fed the NIH31 diet for their entire life. All mice were singly housed in a climate controlled environment with 12:12 light:dark cycle and water was provided *ad libitum*. For all analyses, 2–3 mice per group were analyzed. Due to the closing of the NIA caloric restricted rodent colony, we were unable to obtain more mice for experiments and thus the results presented are preliminary insights into the ability of CR to modulate gut microbiota and influenza responses with aging. All procedures were approved by the University of Connecticut School of Medicine Institutional Animal Care and Use Committee and carried out in accordance with these regulations. All mice underwent gross pathological examination at time of sacrifice and animals with obvious pathology were excluded from the study.

### Viral Infection and Analysis

To infect with Influenza virus A/PR/8/34 (PR8), 400 EID_50_ were given intranasally in 40 μl to isoflurane anesthetized animals. Mice were sacrificed 7 days post infection. Lungs were harvested and the viral burden in mRNA from digested whole lung tissue was determined by real-time PCR measuring influenza *polymerase acidic protein* gene (PA) copy number (74, 75). BAL was collected by flushing lungs with 1 ml saline. BAL and serum were assayed for cytokine and chemokine content using the Luminex Mouse Cytokine/Chemokine 32-plex panel (EMD Millipore, Billerica, MA, USA).

### Stool Collection and Microbiome Analysis

Fecal samples were collected between 6:00 a.m. and 7:00 a.m. in the morning each day beginning 3 days prior to infection (day −3) and stored at −80°C immediately after collection for microbiome analysis. A total number of 187 samples were collected. Total DNA was extracted from fecal samples by using Power Soil DNA Extraction kit (Mo Bio Laboratories, Carlsbad, CA, USA) per manufacturer’s protocol. Bacterial 16S ribosomal RNA gene was amplified by using the 27F/534R primer set (27F 5′-AGAGTTTGATCCTGGCTCAG-3′, 534R 5′-ATTACCGCGGCTGCTGG-3′). PCR reactions were performed using phusion high-fidelity PCR Mastermix (Invitrogen, Carlsbad, CA, USA) with the following condition: 95°C for 2 min (1 cycle), 95°C for 20 s/56°C for 30 s/72°C for 1 min (30 cycles). PCR products were purified using Agencourt AMPure XP beads (Beckman Coulter, Brea, CA, USA) according to manufacturer’s protocol. Library was prepared with Illumina’s instruction specifically for Miseq platform. 17 samples failed the DNA extraction and sequencing library preparation. Full microbiome data are available at https://www.ncbi.nlm.nih.gov/bioproject/PRJNA393321.

### Statistical Analysis

Weight loss and cytokine/chemokine parameters were analyzed *via* two-way ANOVA with Bonferroni *post hoc* corrections (GraphPad Prism, GraphPad Software, Inc., La Jolla, CA, USA). Raw sequencing reads were assembled using FLASH (75). Chimeric sequences were removed using USEARCH (76). Operational taxonomic units (OTUs) were generated at ≥97% sequence similarity. Taxonomic assignment of OTUs was performed by comparing sequences to the RDP database (Confidence threshold = 50%) (77). The R package “phyloseq” (78) was used for alpha diversity and beta dissimilarity analysis. Relationship between microbiota phyla and influenza-induced weight loss and serum/BAL cytokine/chemokine concentration were analyzed *via* Spearman’s correlation using GraphPad software for Mac 6.0 (GraphPad Prism, La Jolla, CA, USA) and R package “corrplot” (79). Corrplot: visualization of a correlation matrix. R package version 0.77.

## Results

The goal of this study was to explore the interaction of aging, diet, and influenza infection with the gut microbiome. We aimed to gain preliminary insight into how aging may impact influenza-induced microbiome changes, as well as how age-related microbiome changes may impact influenza responses. The first part of the study examined how age and CR impact the response to flu, while the second part examined the effect on the composition of gut microbiome. For these studies, we employed a sublethal infection dose and examined immune parameters at 7 days postinfection. At this time point, influenza-induced weight loss becomes more evident, however, is not different between ages (80) or CR and AL groups (unpublished experiments from the Haynes lab). Thus, percent weight loss should not be a confounding factor between groups and should not put CR mice at greater risk to succumb to infection (45, 46). While this limits the observed differences between groups due to infection, it allows us to assess early time points where the gut microbiome may play a role. Additionally, since it is known that weight loss itself affects gut microbiota (81–83), this control was necessary to gain preliminary insight into influenza-induced alterations.

### Effect of Age and CR on the Response to Influenza Infection

**Figure 1:**
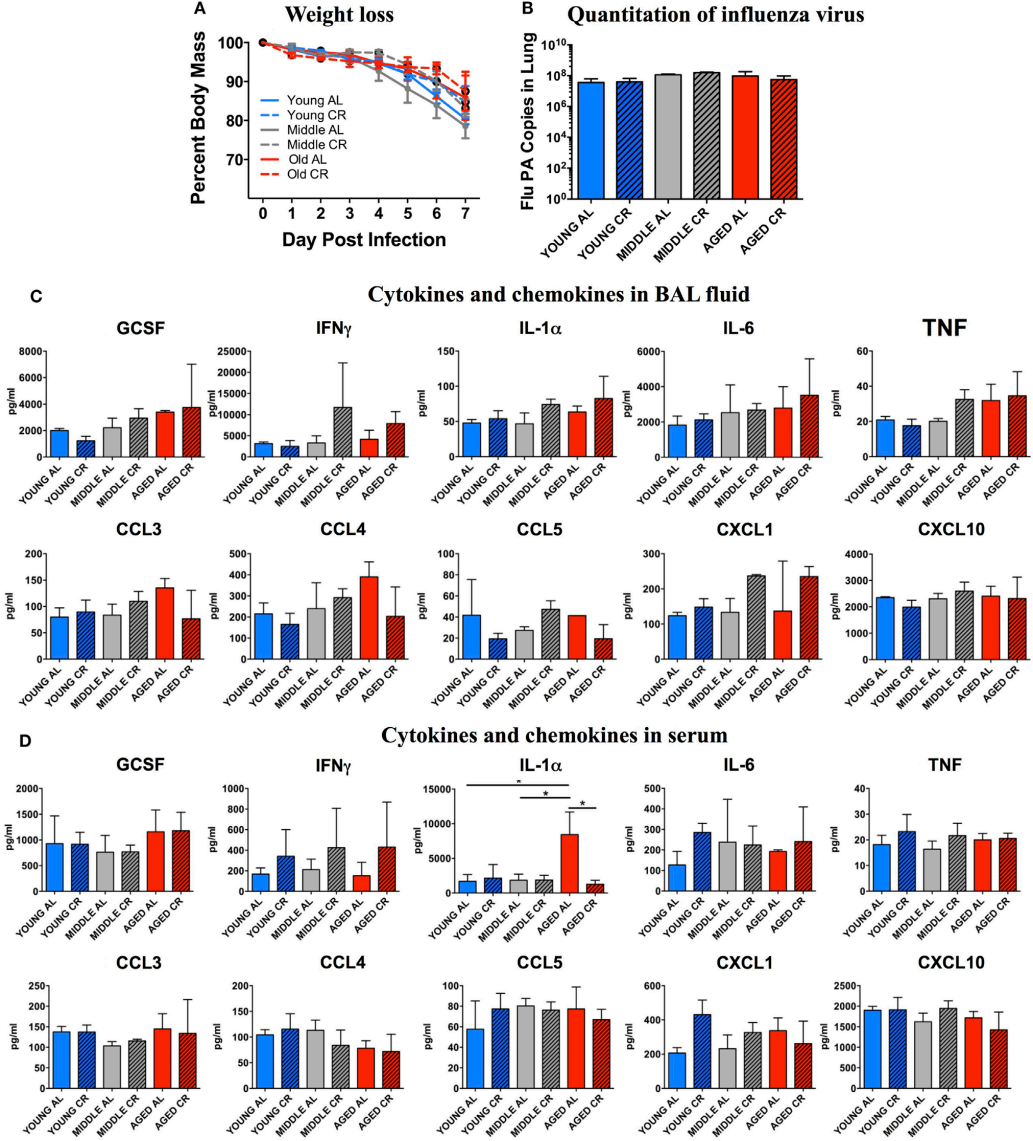
Response to influenza infection. Young, middle-aged, and aged C57BL6 mice on an *ad libitum* (AL) or caloric-restricted (CR) diet were anesthetized and infected with 400 EID50 of influenza virus. **(A)** Each day, mice were weighed and the percent of the starting weight is shown. On day 7 postinfection, mice were sacrificed. **(B)** mRNA was isolated from the lungs and influenza virus was quantitated by real-time PCR measuring influenza *polymerase acidic protein* gene (*PA*) copy number. **(C)** Bronchiolar lavage fluid and **(D)** serum was collected and subjected to multiplex analysis to determine levels of cytokines and chemokines. Data were analyzed *via* two-way ANOVA with Bonferroni *post hoc* corrections; *p < 0.05.

Weight loss was monitored throughout the experiment. Figure 1A shows that there were no significant differences in percent weight loss in any of the groups regardless of age or diet. In addition, Figure 1B shows that on day 7 postinfection, there was no significant difference in the amount of flu virus in the lungs in each group. These correlate well with our previous studies showing that the main age-related differences in response to sublethal influenza infection are not seen during the peak of the infection during the first week, but become apparent during the resolution phase in week two following flu infection (8).

The BAL fluid from each mouse was analyzed for cytokine and chemokine contents. As shown in Figure 1C, there were no significant differences in cytokines important for a protective immune response to influenza infection including GCSF, IFNγ, IL-1α, IL-6, and TNF. In addition, there were no significant differences in chemokines that recruit protective immune cells to the lungs including CCL3, CCL4, CCL5, CXCL1, and CXCL10. A similar pattern was also observed when the serum from these groups was analyzed (Figure 1D) except for a significant increase in IL-1α in the aged AL group that was not seen in aged CR mice. Thus, both locally in the BAL and systemically in the serum, there are few measurable differences between the response to infection in young, middle aged, and aged groups and between AL and CR groups at this relatively early time point following flu infection.

### Effect of Age and CR on the Gut Microbiome during Influenza Infection

**Figure 2:**
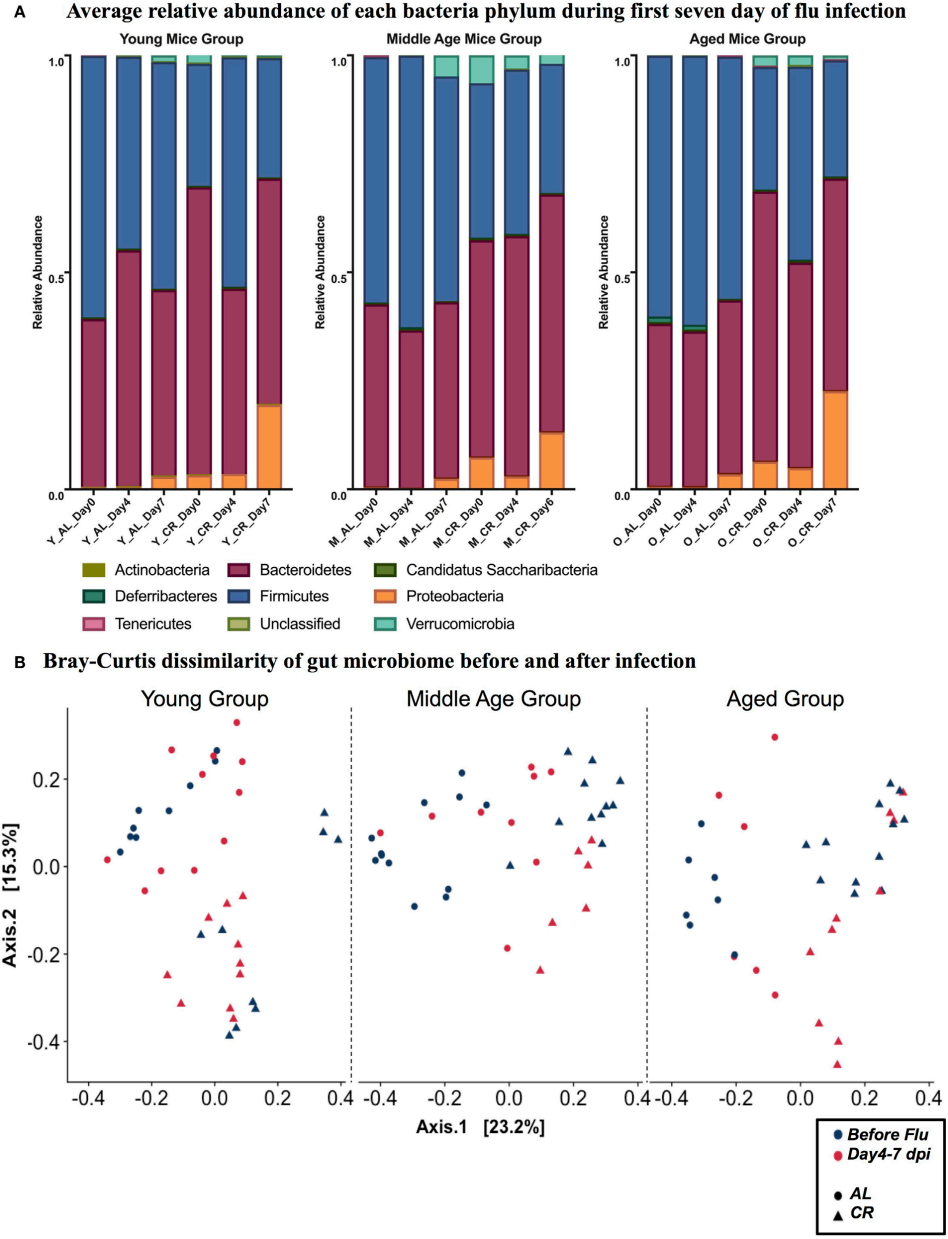
**(A)** Relative abundance of nine phyla at 0, 4, and 7 day postinfection (dpi) in young, middle, and aged *ad libitum* (AL) or caloric-restricted (CR) mice. For middle-aged CR group, we do not have data at 7 dpi, so 6 dpi is shown here instead. Relative abundance of bacteria is shown as a fraction. **(B)** PCoA plot of Bray Curtis dissimilarity prior to (naïve, −3–0 dpi) and after (postinfection, 4–7 dpi) flu infection.

It is known that gut microbiome is affected by influenza viral infection (56). To fully understand the dynamics of this process and its implication, stool samples were collected prior to and during influenza infection. Analysis was done by reconstruction of the gut bacterial microbiome by amplicon sequencing to access the composition of the microbiota population. Figure 2A shows the relative abundance of nine bacterial phyla in each experimental group. While there are no major age-related differences apparent in phylum level, there are differences due to influenza infection and diet. In each age category, the distribution of phyla is changed by day 7 postinfection in both AL and CR groups, characterized by an increase in Proteobacteria and Verrucomicrobia. Furthermore, Proteobacteria and Verrucomicrobia are more abundant in the CR groups when compared to AL in each age category. Figure 2B shows the Bray Curtis dissimilarity of samples indicating they segregate by diet but not by infection in each age group, this implies that diet had a greater impact on the composition of gut microbiome when compared to the impact of influenza infection.

**Figure 3:**
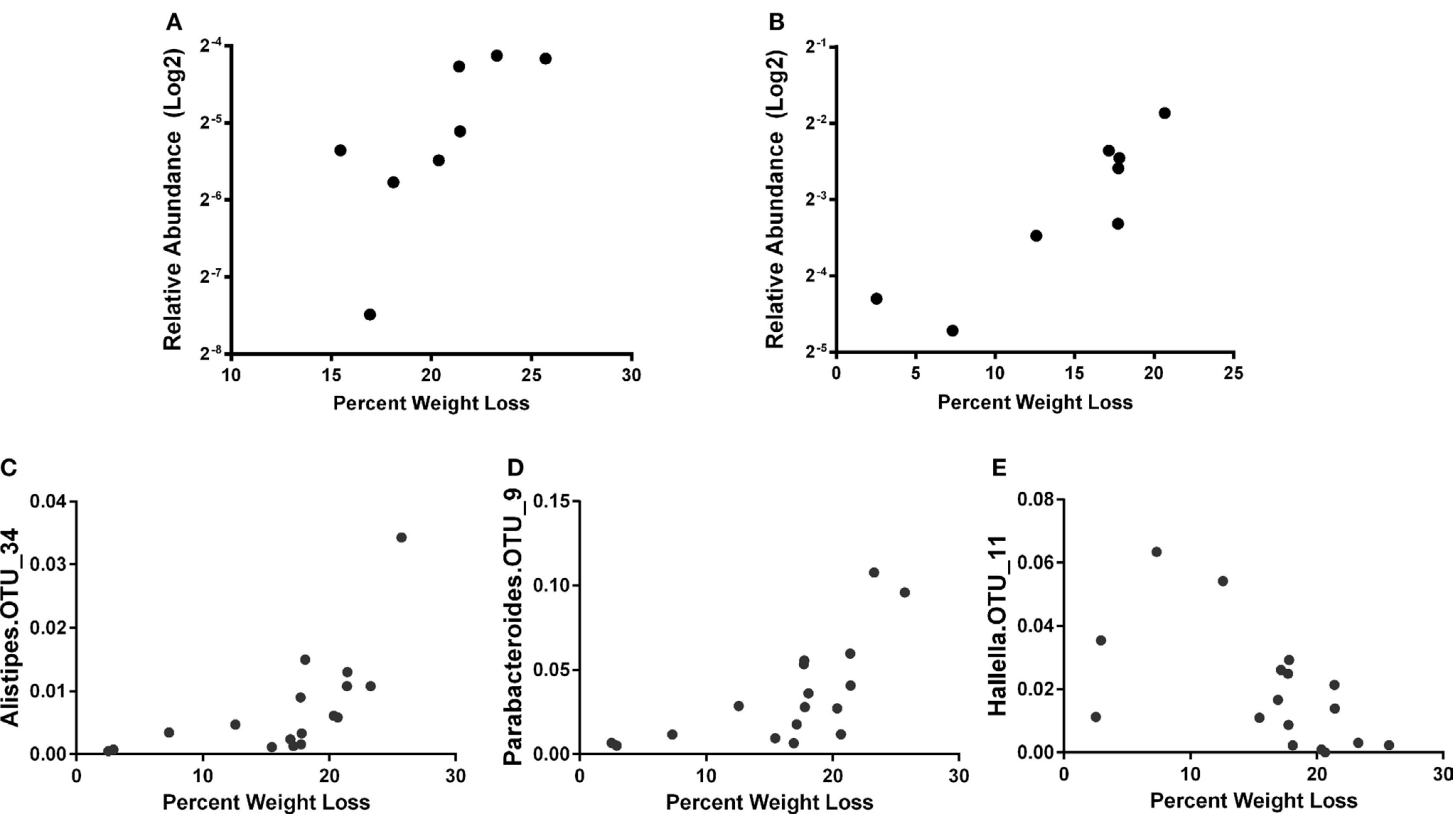
The percent weight loss for AL **(A)** and caloric restricted **(B)** at 7 days post infection (dpi) is plotted against the relative abundance of phylum Proteobacteria (after log2 transform). Correlation of relative abundance of Alistipes OTU_34 **(C)**, Parabacteroides OTU_9 **(D)**, and Hallella OTU_11 **(E)** with percent weight loss among all groups is shown. The bacteria abundance is the average of 5, 6, and 7 dpi. Spearman’s correlation with *p* < 0.05.

**Figure 4:**
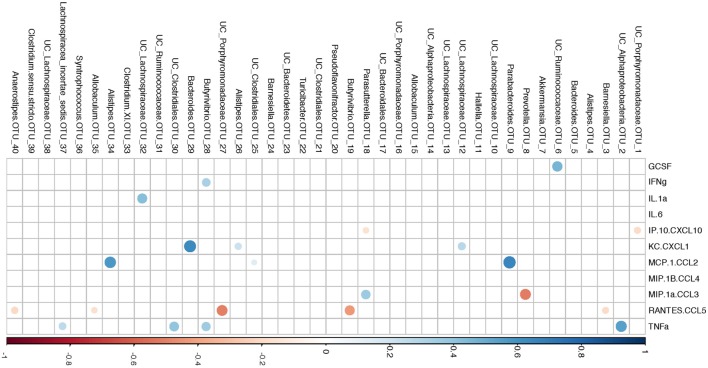
Correlation of operational taxonomic units (OTUs) relative abundance with serum cytokines at 7 day postinfection (dpi). OTUs relative abundance is the average of 5, 6, and 7 dpi. Color intensity and size are proportional to the spearman correlation coefficient (*p* < 0.05). Positive correlations are displayed as blue and negative correlations are displayed in red. UC: unclassified according to RDP database (confidence threshold = 50%).

Similar to a previous report (56), we observed increased phylum Proteobacteria in all groups at day 7 post influenza infection. We determined that regardless of age, the relative abundance of phylum Proteobacteria was positively correlated with percent body weight loss at this time in AL and CR (*r* = 0.8095, *p* = 0.0218 and *r* = 0.8333, *p* = 0.0154; respectively, Figures 3A,B) indicating a relationship between severity of infection and Proteobacteria abundance. To further examine the relationship between flu pathogenicity and gut microbiota abundances, we examined this relationship among all groups and major OTUs. This approach increases the overall sample size (*n* = 17) to increase the power of our correlation analysis and provide preliminary insight into potential key bacteria associated with flu responses regardless of age and diet. Ten specific OTUs correlated with percent weight loss, and interestingly, Alistipes OTU 34 and Parabacteroides OTU 9, both members of the Bacteroidetes phyla showed the strongest relationship (*r* = 0.8235, *p* < 0.0001 and *r* = 0.7672, *p* = 0.0005; respectively, Figures 3C,D), while another Bacteroidetes, Hallella OUT_11 was the next strongest relationship, however, was negatively correlated with percent weight loss (*r* = −0.5907, *p* = 0.0216, Figure 3E); highlighting differential relationships within phyla. Next, we examined the relationship between serum cytokine/chemokines and major OTUs observed among all groups to determine how influenza-induced inflammation relates to bacterial abundances (Figure 4). We observed 22 significant correlations between relative bacterial abundance and inflammatory mediators in the serum. Of note, CXCL1 was positively correlated with unclassified Lachnospiraceae OTU_12, Alistipes OTU 26, and Bacteroides OTU_29, CCL2 was positively correlated with Parabacteroides OTU_9, Alistipes OTU_34, and Unclassified Clostridiales OTU_25, and TNFα was positively correlated with unclassified alpha-Proteobacteria OTU_2, Butyrivibrio OTU_28, Unclassified Clostridiales OTU_30, and lachnospiraceae incertae sedis OTU_37. Conversely, CXCL10 was negatively correlated with Unclassified Porphyromonadaceae OTU_1 and Parasutterella OTU_18, and CCL5 is negatively correlated with Barnesiella OTU_3, Butyrivibrio OTU_28, Unclassified Porphyromonadaceae OTU_27, Allobaculum OTU_35, and Anaerostipes OTU_40. Among those correlations we observed, the three strongest positive correlations were CXCL1 with Bacteroides OTU_29, CCL2 with Parabacteroides OTU_9, and CCL2 with Alistipes OTU_34. The three strongest negative correlation were CCL3 with Prevotella OTU_8, CCL5 with Unclassified Porphyromonadaceae OTU_27, and CCL5 with Butyrivibrio OTU_19. Thus, systemic immune responses were related to gut microbiota alterations. Interestingly, aside from Butyrivibrio, the strongest correlations, both positive and negative, were Bacteroidetes. Though it is not possible to conclude any causal relations between these bacteria taxa and the correlated host response, these findings provide insight into future research in manipulating bacterial gut microbiome to facilitate antiviral immune responses.

## Discussion

In this exploratory study, we sought to obtain preliminary information as to how CR, a known modulator of aging and gut microbiota, could influence influenza-induced gut microbiota changes and immune responses during acute influenza infection in young, middle aged, and aged mice. Different outcomes of influenza infection, due to the influence of aging and CR, could be mediated by modulation of the gut microbiota by these factors with subsequent effects of infection due to the different microbial communities. We observed that CR affected the gut microbial communities (Figure 2), as found in previous studies, and that age also has an effect (the different patterns in Figure 2B). However, no obvious effect of infection was observed (Figure 2). Unlike previous studies (41, 45), we did not observe significant differences in weight loss or viral burden following influenza infection in CR aged animals. However, our study differed in that we utilized a sublethal dose of influenza and sacrificed mice at a relatively early point of infection, and both of these could contribute to a lessening of the effect of infection on the microbiome.

Although there was not a large effect on microbial community structure following infection, we did observe an increase in the proportion of Proteobacteria, which was more significant in CR mice but independent of age (Figure 2A). The increase in the proportion of Proteobacteria was correlated with weight loss (Figure 3), taken as a marker of infection severity. This again was independent of age. Further, multiple OTUs correlated with weight loss from the bacteroidetes phyla. This connection raises the possibility that a change in the microbiome has a connection with infection outcome, as hypothesized above; however, the relationship does not seem to be straight forward and members of the same phyla have differential relationships. Future research can examine if elimination or transfer of these specific bacteria can impact flu responses. Indeed, others have shown antibiotic treatment is detrimental to flu immune responses, specifically oral neomycin eliminated Gram-positive bacteria and impaired immune responses (53). Here, we identify Gram-negative bacteria that may also be crucial for immune responses.

The PR8 strain of influenza virus used for these studies will not directly infect gut tissue of B6 mice (84), raising the question of how a respiratory infection can affect the gut microbiome. The mucosal surfaces in lung and gut are considered a common mucosal surface that share immunological signals (85), so inflammation from one site is likely to affect the other site. Since Proteobacteria are generally observed during gut inflammation (86), the more severe infection in the lung, the greater the effect on the gut, and this would result in the observed correlation between Proteobacteria (a measure of inflammation in the gut) and weight loss (a measure of infection). Finally, we note that overgrowth of Proteobacteria can be impaired by blocking the type I interferon (IFN-I) signal (56), suggesting this gut microbiota change correlated with lung inflammation is mediated by an IFN-I-related immune response.

The gut is believed to be the largest immunological compartment in the body (87) and thus signaling from the gut microbiome may play an important role in viral infections. For example, mouse mammary tumor virus (88) and Enteric virus (89) require intestinal bacterial flora to establish effective infection. Lymphocytic choriomeningitis virus and influenza virus (90), conversely, will trigger a more effective immune response if intestinal bacterial flora is present. Also, germ-free mice (91), mice treated with an antibiotic cocktail (53), or TLR5 KO mice (with impaired function in sensing bacterial flagellin) (92) will not generate adequate immune response to influenza viral infection. Thus, the microbiome helps protect during flu infection. Although here we show a correlation between Proteobacteria abundance and infection, each member of the microbiota may signal the immune system in a different manner (93). For example, a previous report (53) showed that a host with antibiotic treatment to largely deplete Lachnospiraceae would not generate a good antibody response against trivalent inactivated influenza vaccine. Similarly, we noticed that higher relative abundance of genus Lachnospiraceae OTU_12 and OTU_32 are correlated with higher amount of CXCL1 and IL-1α, respectively. More generally, we show here that cytokine production associated with flu infection in our study correlates differently for each of 22 OTUs (Figure 4). This suggests that members of the microbiome regulate the immune response in different ways.

These preliminary findings contribute to the understanding of dynamics and complexity of gut bacterial microbiota and influenza infection. We are the first to show the early changes (Day 4 post influenza infection) of gut bacterial microbiota composition in both young, middle, and aged mice on both AL or CR diet. Interestingly, though CR had a great impact on gut microbiota, it did not seem to affect flu-induced immune responses or flu-induced alterations in the gut microbiota at these early time points. It is possible that these alterations, such as increased proteobacteria compared to AL mice, may have effects at later time points in flu responses and recovery. More research is necessary to determine if CR modulation of gut microbiota with aging is beneficial to flu immune responses. Cytokine production associated with influenza infection in our study correlates differently with each OTUs. Although the data we present do not allow causal relations between bacteria and cytokine production to be determined, they do provide hypotheses for virus-bacterial interactions through the immune system. There is evidence that the genus *Lactobacillus* can improve the immune response to the PR8 strain of influenza (94, 95) and respiratory syncytial virus (96) infection. Here, we find multiple members of the Bacteroidetes phyla to be correlated with immune parameters and flu pathogenicity. Thus, while influenza infection promotes Proteobacteria overgrowth through IFN-I (56), members of the Bacteroidetes phyla are also affected and likely in turn affect immune parameters, both positively and negatively. Interestingly, a Bacteroidetes dominated microbiome was associated with increased frailty among the elderly (61), perhaps suggesting a microbiota link associated with the increased disability observed following influenza infection in the elderly (97). Future research should explore manipulation of bacterial species from this phyla to modulate flu immune responses. Thus, we believe this exploratory study can provide some additional guidance to the use of microbiota to facilitate virus-specific immune responses, especially for elderly whose immune responses are known to be deficient.

## Ethics Statement

This study was carried out in accordance with all federal, state, and institutional laws, policies, and guidelines. The protocol was approved by the UConn Health Institutional Animal Care and Use Committee.

## Author Contributions

JB. and LH designed the study; JB carried out the influenza infection and analysis of responses; XZ and GW performed the microbiome analysis; JB, LH, GK, XZ, and GW participated in preparation of the figures and manuscript.

## Conflict of Interest Statement

The authors declare that this research was conducted in the absence of any commercial or financial relationships that could be considered a potential conflict of interest.
